# Anti-diabetic effect of red quinoa polysaccharide on type 2 diabetic mellitus mice induced by streptozotocin and high-fat diet

**DOI:** 10.3389/fmicb.2024.1308866

**Published:** 2024-02-27

**Authors:** Yanqing Zang, Yinchen Ge, Yang Cao, Huacheng Tang

**Affiliations:** ^1^College of Food Science and Engineering, Heilongjiang Bayi Agriculture University, Daqing, Heilongjiang, China; ^2^Chinese National Engineering Research Center, Daqing, Heilongjiang, China; ^3^College of Animal Science and Technology, Heilongjiang Bayi Agricultural University, Daqing, Heilongjiang, China

**Keywords:** red quinoa polysaccharides, high-fat diet, physicochemical properties, gut microbiota, antioxidant activity

## Abstract

The purpose of this study was to explore the mechanism of red quinoa polysaccharide (RQP) in alleviating type 2 diabetes (T2D) through *in vivo* and *in vitro* experiments. Results of HPLC and FITR showed that RQP was a complex polysaccharide and contained more glucose, galactose and acarbose. *In vitro* experiments, RQP showed strong antioxidant capacity and inhibition on α-amylase and α-glucosidase. *In vivo* experiments, RQP was proved to induce a significant improvement of diabetes after 4 weeks of ingestion, including the abilities of lowering blood glucose, regulating lipid metabolism, anti-oxidation and promoting secretion of SCFAs. Furthermore, 16S rRNA study demonstrated that RQP transformed the intestinal microbiota composition in diabetic mice, decreased the abundance of *norank_f_Muribaculaceae* and *Lachnospiraceae_NK4A136_group*, and increased the relative abundance of *Akkermansia*, *unclassified_f_Lachnospiraceae*, *norank_f_Eubacterium_coprostanoligenes_group*, *unclassified_f_Atopobiaceae* and *norank_f_Lachnospiraceae*. The biosynthetic pathways, metabolic pathways and intestinal microbiome phenotypes in mice also changed accordingly. In conclusion, this study suggests that RQP can inhibit the development of diabetes by correcting the imbalance of intestinal flora.

## Graphical Abstract

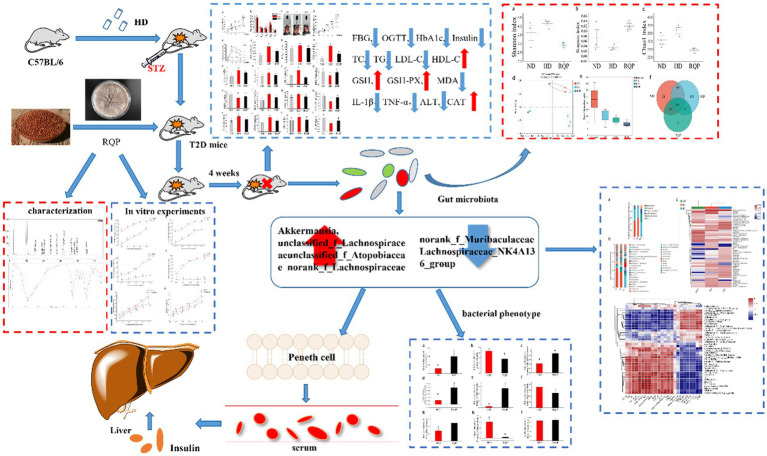

## 1 Introduction

T2D is a serious health problem worldwide. It is defined by insulin insufficiency caused by pancreatic beta cell failure and insulin resistance in target organs (Chatterjee et al., 2017). The data showed that increased availability of high-calorie foods and decreased physical activity have led to lifestyle changes, that have resulted in a global increase in T2D and prediabetes (Saeedi et al., 2019). The International Diabetes Federation (IDF) Diabetes Map 2021, 10th edition, 537 million people have diabetes globally in 2021, and it will reach 783 million by 2045 (Magliano and Boyko, 2021). Researchers around the world are currently focusing on the causes, prevention, and treatment of T2D. Therefore, in this context, the beneficial role of polysaccharides in plant extracts in lowering blood glucose and inhibiting the development of diabetes mellitus has received extensive attention by researchers.

Quinoa (*Chenopodium quinoa* Wild, family Amaranthaceous) is a pseudo-cereal from the Andean region that was once called the “mother of grains” by the Incas and is now becoming increasingly popular because of its great nutritional value (Campos et al., 2018). Quinoa is rich in protein and has a balanced ratio of amino acids, with a nutritional value like milk, and is easily absorbed by the body (Ren et al., 2023). Studies have shown that quinoa also contains many biologically active substances such as flavonoids and polysaccharides. These substances have been shown to prevent many diseases such as cancer, inflammatory disease, and cardiovascular diseases (Ren et al., 2023). Some studies have proved the biological activities of quinoa polysaccharides (Cao et al., 2020). However, there are few reports on the health function of red quinoa polysaccharide.

According to a recent study, alterations in the composition of the gut microbiota are linked to the emergence of diabetes and are related to the other metabolic diseases (Salgaco et al., 2019). Numerous studies have demonstrated that controlling the intestinal microbiota of the host can reduce the symptoms of diabetes (Cani et al., 2008; Gurung et al., 2020). The host’s energy homeostasis can be affected by gut microbiota through the method of altering the number of active substances, which in turn controls the production of insulin (Wu et al., 2021). The influence of red quinoa polysaccharide on modifying the make-up of the gut microbiota has not yet been properly clarified. We investigated whether red quinoa polysaccharide has hypoglycemic and lipid-lowering effects on C57BL/6 mice fed a high-fat diet (HD) in the current study.

## 2 Materials and methods

### 2.1 Materials

Red quinoa seeds were obtained from the Hexi Farm (Golmud City, Qinghai Province, China). We purchased 21 male C57BL/6 mice which were maintained in a specific-pathogen-free (SPF) place from Changsheng Biotechnology Co., Ltd. (Liaoning Province, China). Normal diet (ND) and high-fat diet (HD) were provided from Maohua Biotechnology Co., Ltd. (Liaoning Province, China). Assay kits were used to assess the serum’s biochemical characteristics. Assay kits for total cholesterol (TC), triglyceride (TG), low density lipoprotein cholesterol (LDL-C), high density lipoprotein cholesterol (HDL-C), alpha-alanine aminotransferase (ALT), catalase (CAT), glycosylated hemoglobin (HbA_1c_), insulin (INS), Tumor Necrosis Factor-α (TNF-α), interleukin -1β (IL-1β), nitric oxide (NO), reduced glutathione (GSH), glutathione peroxidase (GSH-PX) and malondialdehyde (MDA) were purchased from Nanjing Jiancheng Bioengineering Institute (Nanjing, Jiangsu Province, China). All other chemicals and reagents are analytically pure.

### 2.2 Preparation of RQP

The extraction and purification of polysaccharides from red quinoa was performed according to the methods described by Hu with a slight modification (Hu et al., 2017). Seeds of quinoa were ground into powder. The powder was passed through an 80-mesh sieve and extracted with petroleum ether (1:10, v/v) for 8 h to remove lipids and pigments. The red quinoa powder was then extracted using ultrasonic-assisted extraction technology and distilled water (250 w, 40 KHz). The ultrasonic process took place at a temperature of 60°C for 51 min with a water to material ratio of 20 mL/g. Centrifuge and collect the supernatant, adjust the pH of the supernatant to 8, add protease, bathe in water at 37°C for 30 min, then bathe in water at 90°C for 30 min, cool and adjust the pH to 4.5, and place for 12 h.

Subsequently, the extract was centrifuged at 4,000 × g for 20 min and adjusted to pH 7. The supernatant was then reduced using a rotary evaporator to one tenth of its volume at 60°C and precipitated with 95% ethanol (1:4, v/v) for 12 h at room temperature. Centrifugation was used to gather the resultant precipitate, which included crude polysaccharides. The unprocessed polysaccharide was placed into the dialysis bag for 24 h of dialysis, and throughout that time, the pure water was changed frequently to remove other pollutants. The red quinoa polysaccharide was stored at −20°C after freeze drying.

### 2.3 Characterization of RQP

The structure of RQP was analyzed by FTIR spectrometer. The monosaccharide composition of RQP was measured by HPLC method (Tan et al., 2021). Briefly, RQP was dissolved in 3.0 mL of 2.0 M trifluoroacetic acid (TFA) and hydrolyzed at 120°C for 4 h in sealed glass tubes, respectively. After 4 h, the methanol was added and blow dry with nitrogen to completely remove the remaining TFA and then redissolve it in 3.0 mL of water. The 250 uL of RQP solution was mixed with 250 uL of 0.60 mol/L NaOH and 500 uL of 0.40 mol/L PMP-methanol in 5.0 mLEP tube. Then, the reaction was done at 70°C for 1 h and cooled in water for 10 min. The 500 uL of 0.30 mol/L HCL was added to neutralize and the 1.0 mL of chloroform was added to vortex for 1.0 min and centrifuge at 3,000 r/min for 10 min. The supernatant was extracted three times. The obtained supernatant was put into HPLC for determination.

### 2.4 *In vitro* experiments

#### 2.4.1 *In vitro* antioxidant of RQP

The scavenging and reducing abilities of RQP on DPPH (1,1-diphenyl-2-picryl-hydrazyl radical), ABTS [2,2′-azino-bis (3-ethylbenzthiazoline-6-sulfonic acid)], OH (hydroxyl free radical), and O_2_^−^ (superoxide anion) at various doses (0.20, 0.40, 0.60, 0.80, and 1.0 mg/mL, respectively; Ke et al., 2020). The vitamin C (VC) were determined as a comparative to aid with comprehension. The IC50 value, which was inversely associated with antioxidant activity and used to measure antioxidant activity. Each experiment was repeated three times to take the average value.

Inhibition of DPPH by RQP. The RQP solution 2.0 mL (0.20, 0.40, 0.60, 0.80, and 1.0 mg/mL) were mixed with 2.0 mL DPPH solution (2.0 × 10^−4^ mol/L, dissolved in anhydrous ethanol). The reaction was done for 20 min without light, and the absorbance was measure at 517 nm (A1). Anhydrous ethanol was used instead of DPPH as a control group (A2). Pure water was used as a blank group instead of polysaccharide samples (A0).


$$\mathrm{The}\;\mathrm{clearance}\;\mathrm{of}\;\mathrm{DPPH}\;\left(\%\right)=1-\left[\left(A1-A2\right)/A0\right]\times 100$$

Inhibition of ABTS by RQP. The 0.20 g ABTS and 0.0344 g potassium persulfate were mixed in 52 mL of distilled water. Then stored at room temperature in the dark for 24 h as ABTS mother liquor and was measured at 734 nm (0.70 ± 0.02). 0.4 mL of RQP solution with different concentrations was taken from each test tube, and then 3.6 mL of ABTS solution was added and the reaction took 15 min at room temperature. The absorbance of samples with different concentrations was measured at 734 nm (A1). Distilled water is added to the RQP solution instead of the ABTS solution (A2). The absorbance of blank solution (A0).


$$\mathrm{The}\;\mathrm{clearance}\;\mathrm{of}\;\mathrm{ABTS}\;\left(\%\right)=1-\left[\left(A1-A2\right)/A0\right]\times 100$$

Inhibition of ·OH by RQP. The RQP solution 1.0 mL (0.20, 0.40, 0.60, 0.80, and 1.0 mg/mL) were added with 1.0 mL 5 mmol/L FeSO_4_, salicylic acid and H_2_O_2_ solution, respectively. The reaction was carried out at 37°C for 30 min, and the absorbance at 510 nm was measured (A1). Distilled water was added to the RQP solution instead of the H_2_O_2_ solution (A2). The absorbance of blank solution (A0).


$$\mathrm{The}\;\mathrm{clearance}\;\mathrm{of}\cdot OH\;\left(\%\right)=1-\left[\left(A1-A2\right)/A0\right]\times 100$$

Inhibition of O_2_^−^ by RQP. The 5.0 mL 0.05 mol/L Tris–HCL buffer (pH 8.2) in a water bath at 25°C for 20 min. The above different concentrations of RQP solution 1.0 mL and pyrogallol solution 1.0 mL were added and shaken. The reaction was carried out in a water bath at 25°C for 5 min. Then, the reaction was terminated by adding 1 mL of 10 mol/L HCL, and the absorbance was measured at 320 nm (A1). Distilled water was added to the RQP solution instead of the o-toluene trios’ solution (A2). The absorbance of blank solution (A0).


$$\mathrm{The}\;\mathrm{clearance}\;\mathrm{of}\;{O_{2}}^{-}\;\left(\%\right)=1-\left[\left(A1-A2\right)/A0\right]\times 100$$

#### 2.4.2 Effects of RQP on inhibition of α-amylase and α-glucosidase

The previously published method was used to assess the α-amylase inhibitory activities of RQP at various concentrations (0.10, 0.20, 0.40, 0.60, 0.80, and 1.0 mg/mL) with a few minor modifications (Lv et al., 2021). Acarbose was selected as the positive control. Each experiment was repeated three times to take the average value.

Preparation of α-amylase and starch with phosphate buffer solution (0.10 mol/L PBS, pH 6.8). 0.50 mL of α-amylase (1 U/mL) and 1.0 mL of RQP at different concentrations were added to the test tubes and incubated for 10 min at 37°C. Then 2.5 mL of starch solution (10 g/L) was added and incubated for 8 min at 37°C. The 1.0 mL of DNS developer was added in the test tubes and incubated for 8 min at 95°C. After cooling to room temperature, the appropriate amount of distilled water was added to dilute and measured the absorbance value at 540 nm. The following formula was used to calculate the inhibitory activity of α-amylase:


$$\mathrm{Inhibition}\;\mathrm{rate}\;\left(\%\right)=\left[\left(A2-As+\mathrm{Ab}\right)/\left(A2-A1\right)\right]\times 100$$

Where As was the absorbance of the test sample system, Ab was the absorbance of the test sample system without the enzyme solution, and A1 and A2 were the absorbances of the starch system and the starch system, respectively.

The polysaccharide’s ability to block α-glucosidase was tested using this methodology, with a few minor modifications (Lv et al., 2021). The 2.0 mL of RQP solution (0.10, 0.20, 0.40, 0.60, 0.80, and 1.0 mg/mL) were mixed with 2.0 mL of 0.10 mol/L phosphate buffer solution (pH = 6.8) and 1.0 mL of 1.0 U/mL of α-glucosidase, respectively. The reaction was warmed in a water bath at 37°C for 15 min. 2.0 mL of 1 mmol/L PNPG was added and mixed thoroughly. 37°C water bath was used for 20 min. 3.0 mL of 0.20 mol/L Na_2_CO_3_ was added to terminate the reaction. The absorbance was measured at 405 nm. The formula below was used to calculate the inhibitory activity of α-glucosidase:


$$\mathrm{Inhibitory}\;\mathrm{rate}\;\left(\%\right)=\left[1-\left(As-\mathrm{Ab}\right)/A0\right]\times 100$$

Where As, Ab, and A0 stood for the absorbance of the test sample system, a mixture of polysaccharide and PNPG without an enzyme, and a combination of PNPG and an enzyme without a mixture of polysaccharide, respectively.

#### 2.4.3 Simulated digestion of RQP

The simulated digestion of RQP was performed as the procedures described in the literature with minor modifications (Ma et al., 2022). RQP was reacted with saliva to simulate oral digestion (0, 1, and 2 h), and mixed with simulated gastric to reacted (0, 2, 4, and 6 h). The liquid after digestion of simulated gastric juice for 6 h was collected and added to simulated intestinal fluid to reacted (0, 2, 4, and 6 h). The samples collected at different time periods were inactivated in a boiling water bath for 5 min to determine the contents of reducing sugar.

### 2.5 Animal experiment

#### 2.5.1 Experimental design

Animal experiments carried out in this study have complied with the ARRIVE guidelines and the U.K. Animals (Scientific Procedures) Act, 1986, EU Directive 2010/63/EU for animal experiments, and the National Research Council’s Guide for the Care and Use of Laboratory Animals and were approved by the Experimental Animals Ethics Committee of Heilongjiang Bayi Agricultural University (Laboratory Animal Approval Number: SPXY2023001). 6 weeks old, 21 ± 1 g, specific pathogen-free male C57BL/6 J mice were subjected to the treatment after a week of acclimatization to the lab environment. The mice were kept in an animal home with free access to food and water under regulated circumstances (temperature: 22°C ± 2°C, relative humidity: 45% ± 5%, and a 12 h light/12 h dark cycle). Seven mice in the ND group were fed a standard pellet diet. Mice in the HD group (14 mice) were fed a high-fat diet (10% lard, 20% sucrose, 1% cholesterol, 0.2% sodium deoxycholate and 68.8% standard pellets) to induce diabetes in the mice. After 4 weeks, all mice were fasted overnight and given free access to water. Fresh STZ solution (0.10 mol/L citrate buffer, pH 4.5) was intraperitoneally given into the HD mice at a dose of 30 mg/kg body weight (Jiang et al., 2013; Zang et al., 2023). Citrate buffer was administered into mice in ND. After 72 h, the levels of fasting blood glucose (FBG) were measured using a glucometer (Sinocare, Changsha, China). Diabetic mice were defined as those with FBG levels over 11.1 mmol/L (Lan et al., 2023; Zhao et al., 2023).

The diabetic mice were then randomly split into two groups: HD with daily administration of dosage (800 mg·kg^−1^·day^−1^) red quinoa polysaccharide, and model control group with high-fat diet (Wang et al., 2016; Mao et al., 2020). After 12 h without food but with unlimited access to water, all mice had an anesthetic for anatomy after 4 weeks. Body weight, fasting blood sugar, and food intake were all monitored on a weekly basis. In the last day of treatment, after anaesthetized for anatomy the serum was separated from the abdominal aorta and centrifuged (4°C, 3,500 r/min, 10 min). For further study, liver and colon contents were immediately frozen in liquid nitrogen and transferred to a temperature of −80°C for storage.

#### 2.5.2 Determination of serum and liver biochemical parameters

Serum markers (TC, TG, LDL-C, HDL-C, INS, HbA_1c_, TNF-α, IL-1β) and markers in liver homogenate (GSH-PX, GSH, MDA, NO, ALT, CAT) were measured with the kit.

#### 2.5.3 Determination of SCFAs in cecal contents

The content of SCFAs were determined by high performance liquid chromatography according to the method of Guo et al. (2022).

### 2.6 Gut microbiota detected by 16S rRNA gene sequencing technology

The DNeasy PowerSoil kit (QIAGEN) was used to extract total genomic DNA from fecal samples. Using Nanodrop (Thermo Scientific, NC2000) and agarose gel electrophoresis, the quantity and purity of DNA were verified. Then, using PCR as described in the prior study, the microbiota V3-V4 hypervariable areas of the 16S rRNA gene were amplified. Primer 338F 5-ACTCCTACGGGAGGCAGCAG-3 and Primer 806R 5-GGACTACHVGGGTWTCTAAT-3 were used in the current investigation. A six-base sequence that is exclusive to each sample makes up the barcode. The DNA libraries were created using the TruSeqTM DNA Sample Prep Kit after the PCR products were separated by gel electrophoresis, purified with the AxyPrep DNA Gel Extraction Kit (Axygen, AP-GX-500), and purified. For the library’s quality assurance, the Agilent 2100 Bioanalyzer System (High Sensitive DNA Chip) was utilized. On the Illumina MiSeq platform, the pooled DNA product was paired-end sequenced in accordance with the recommended procedures.

After passing quality control, the Illumina MiSeq sequences were used in the analysis and uploaded to QIIME (Quantitative Insights into Microbial Ecology, v1.9.1) for additional research. The Simpson index, Chao1 index, and Shannon diversity index were calculated by Mothur (v1.30.2) using the operational taxonomy units of representative sequences and their relative abundance. The principal coordinates analysis (PCoA) with weighted and unweighted UniFrac analysis in R software was used to determine the abundance and diversity of the communities among the normal group, high fat group, and red quinoa polysaccharide. Then, using the PCoA scores, MANOVA was used to evaluate the statistical significance of the difference between the groups. According to the LEfSe approach, which employed the Kruskal-Wallis and Wilcoxon rank sum test (*P*<0.05), mothur was utilized to identify significant communities that were crucial for differentiating the various treatment groups. The main communities of the gut microbiota and the serum and liver index were correlated using the Spearman analysis with a threshold *p*-value of 0.05.

### 2.7 Statistics and analysis

The software Origin 2022 (Origin Lab Corporation, Northampton, MA) was used to draw every single figure. Data from repeated measures were analyzed in SPSS 26 using one-way analysis of variance and Tukey’s tests.

## 3 Results

### 3.1 Characterization of RQP

**Figure 1:**
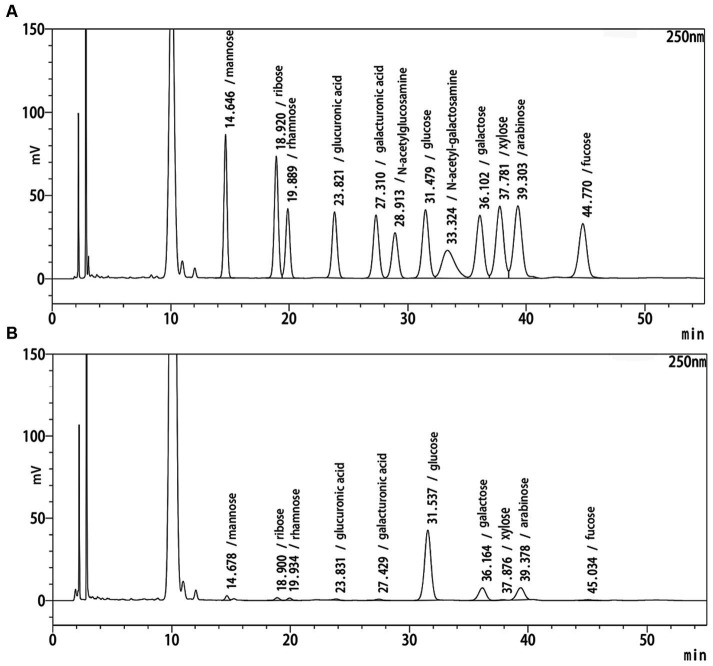
HPLC spectra of RQP. **(A)** monosaccharide standards; **(B)** RQP.

The yield of RQP is 1.57 ± 0.38%. Figures 1A,B show the HPLC chromatograms of the monosaccharide standards and RQP, respectively. The HPLC chromatogram in Figure 1B revealed that RQP was made up of 10 monosaccharides. Among them, glucose, galactose, and arabinose accounted for more than 90% of the monosaccharide composition of RQP (Table 1).

**Table 1:** Monosaccharide composition.

Monosaccharide composition	Sample (%)	
	Monosaccharide standards	RQP
Man	8.950	2.174
Rib	9.608	1.758
Rha	5.748	1.371
GlcA	6.534	0.987
GalA	7.133	1.212
Nag	5.569	-
Glu	8.881	64.545
Nad	7.160	-
Gal	9.195	13.217
Xyl	10.959	0.543
Ara	11.694	13.153
Fuc	8.569	1.040

Man, mannose; Rib, ribose; Rha, rhamnose; GlcA, glucuronic acid; GalA, galacturonic acid; Nag, N-acetyl-glucosamine; Glu, glucose; Nad, N-acetyl-galactosamine; Gal, galactose; Xyl, xylose; Ara, arabinose; Fuc, fucose.

**Figure 2:**
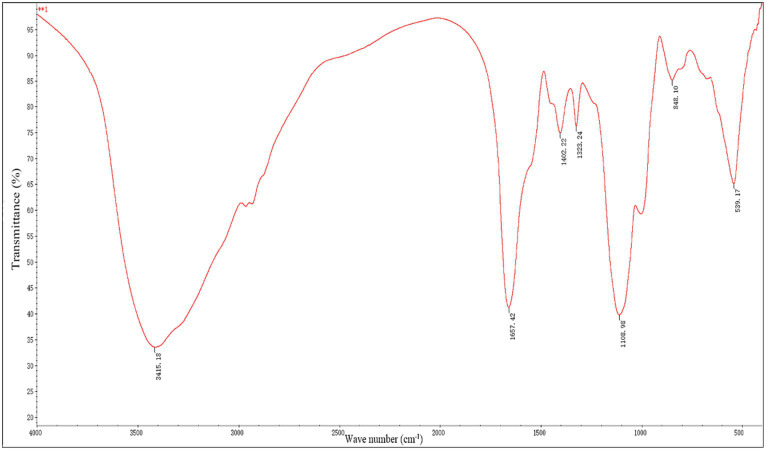
FTIR spectra of RQP.

Figure 2 displays the FTIR spectroscopy data used to describe RQP. The spectra showed polysaccharide absorption peaks around 800–1,200, 1,300–1,800, and 3,200–3,600 cm^−1^. At 3415.18 cm^−1^, the hydroxyl group (O-H) displayed a recognizable broad stretch peak (Li et al., 2021). The presence of uronic acid in RQP can be inferred from the strong band at 1657.42 cm^−1^ and the weak band at 1402.2 cm^−1^ caused by the absorption of a carboxylic group (COO−; Ye et al., 2021). The frequency band at 1330.9 cm^−1^ is caused by the stretching vibration of C = O (Li et al., 2021). The 950–1,200 cm^−1^ FITR range that is utilized to pinpoint the location and strength of unique bands in polysaccharides (Ye et al., 2021). The band at 1108.98 and 848.10 cm^−1^, respectively, verified the presence of pyranose rings and a-glycosidic connections in the monosaccharide blocks of RQP (Portincasa et al., 2022).

### 3.2 *In vitro* experiments

#### 3.2.1 *In vitro* antioxidant

**Figure 3:**
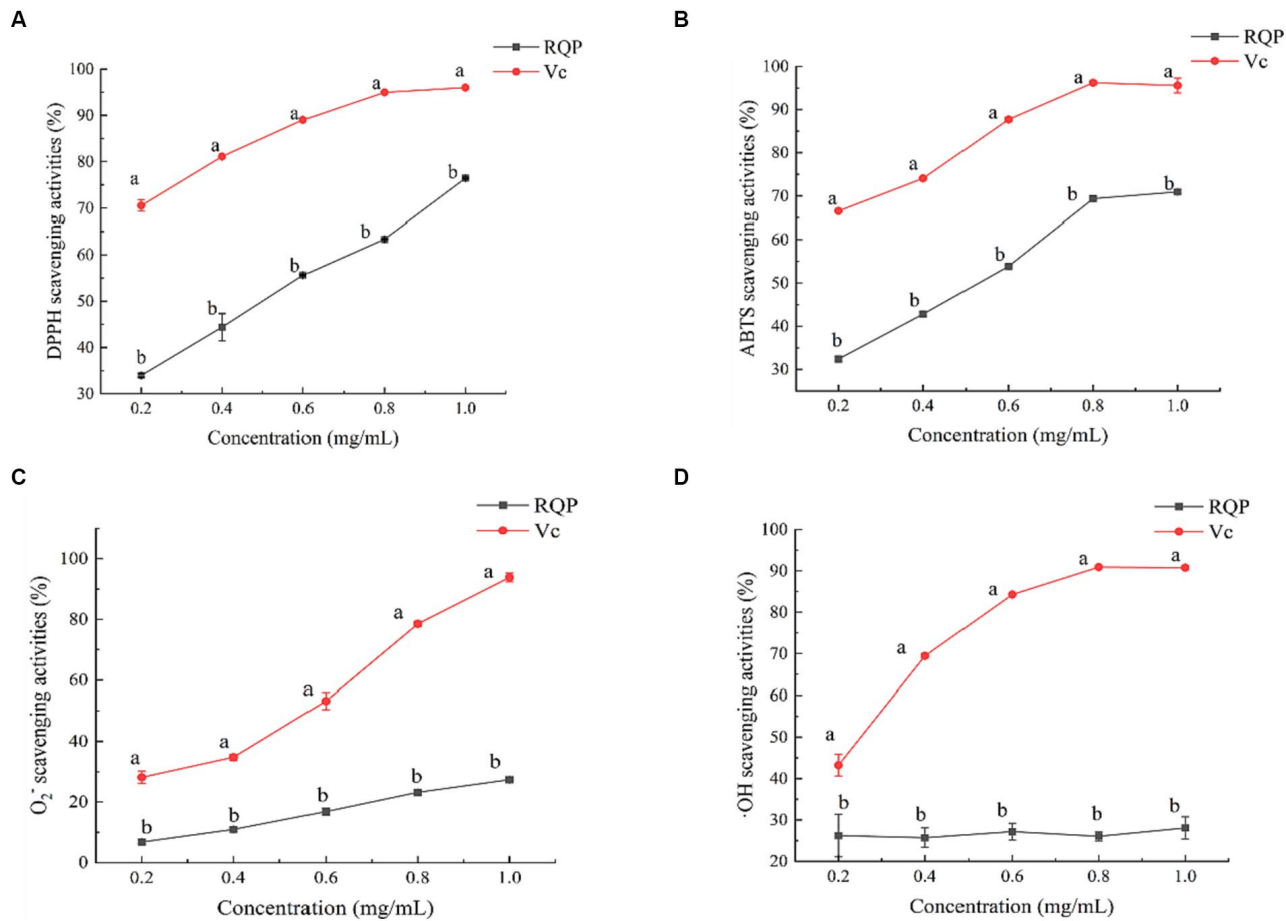
Antioxidant activities of RQP. **(A)** DPPH; **(B)** ABTS; **(C)** O_2_^−^; **(D)** OH. The values are expressed as means ± SD (*n* = 3). Data followed by the different letters are significantly different (*p* < 0.05) by Duncan’s test.

As shown in Figure 3, RQP had strong scavenging ability of DPPH and ABTS free radicals. In terms of DPPH and ABTS scavenging ability, the IC50 values of RQP were 0.51 mg/mL and 0.52 mg/mL. Furthermore, RQP have lower scavenging ability of O_2_^−^ and ·OH, when compared to the VC group.

#### 3.2.2 Inhibition of α-amylase and α-glucosidase by RQP and *in vitro* digestion of RQP

**Figure 4:**
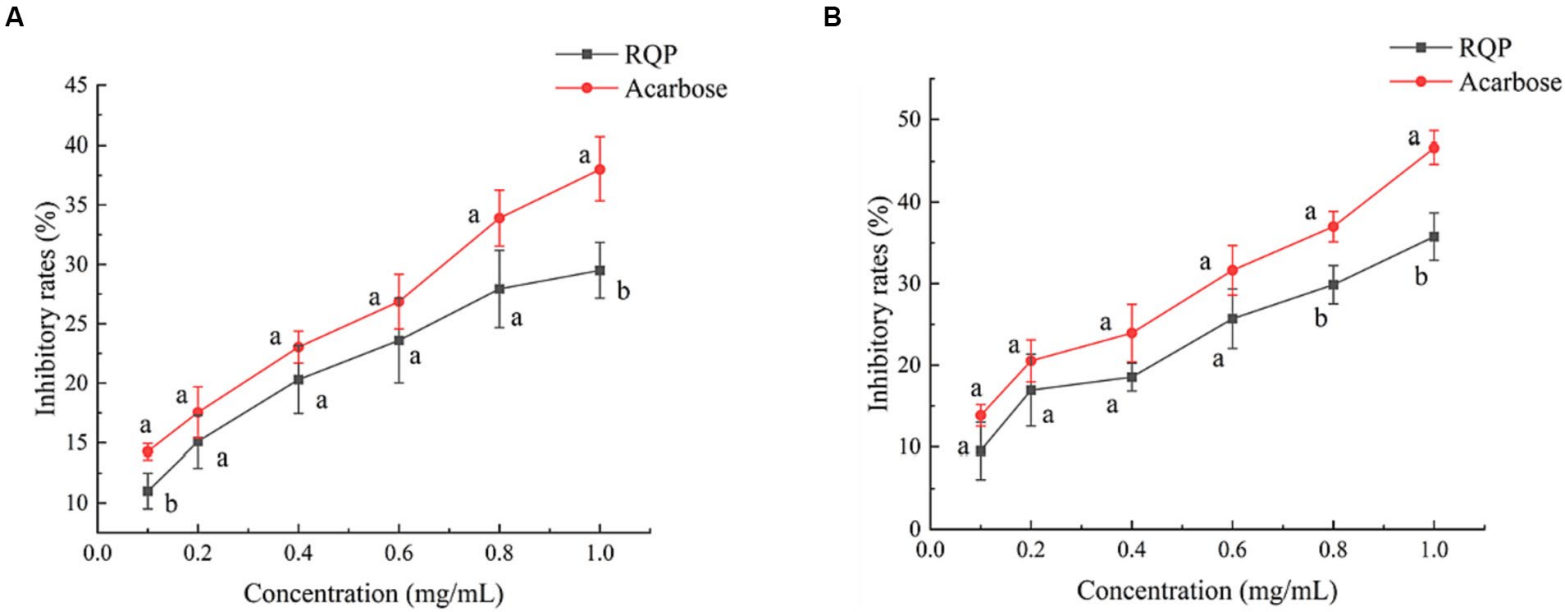
Inhibition on α-amylase **(A)** and α-glucosidase **(B)** activity of RQP. The values are expressed as means ± SD (*n* = 3). Data followed by the different letters are significantly different (*p* < 0.05) by Duncan’s test.

As shown in Figure 4, The inhibition rates of RQP on the activities of α-amylase and α-glucosidase increased with the increase of its concentration in a dose-dependent manner. When the concentration of polysaccharide was 1.0 mg/mL, The inhibition rates of RQP on α-amylase and α-glucosidase were 29.50% and 35.77%, respectively.

Through the preliminary experiment, we got the standard curve of reducing sugar: y = 1.5151x − 0.0135 (R^2^ = 0.9991). To further explore the changes of reducing sugar contents in RQP during digestion include oral cavity, stomach, and small intestine (Supplementary Table S1). As shown in Supplementary Table S1, RQP were not digested in the mouth, stomach, and small intestine. RQP can maintain its original morphology and interact with intestinal microorganisms in the large intestine.

### 3.3 Animal experiments

#### 3.3.1 Effects of RQP on mice body weight, organ index, FBG, OGTT, HbA1c and insulin level

**Figure 5:**
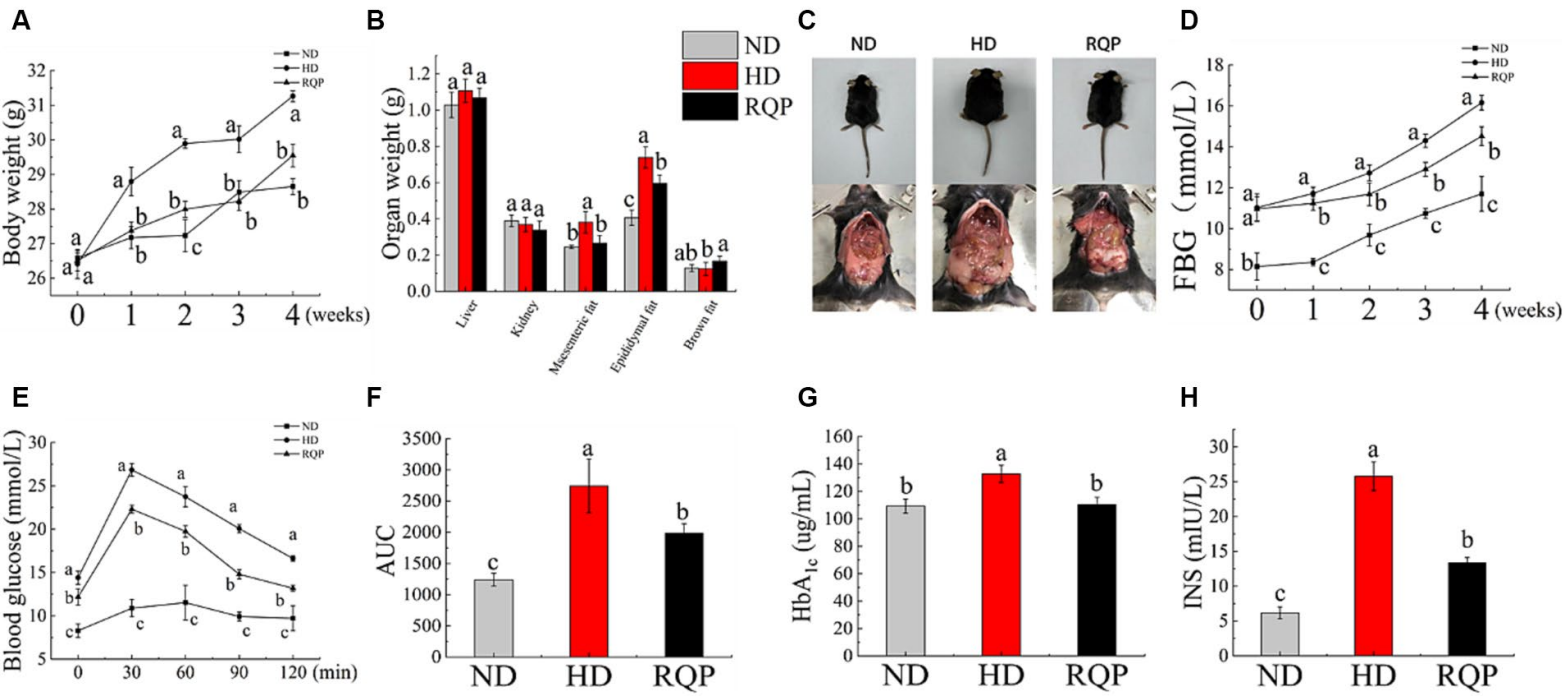
Effects of RQP on diabetic mice: **(A)** body weight; **(B)** organ weight; **(C)** representative images of mice; **(D)** FBG; **(E)** OGTT; **(F)** AUC; **(G)** HbA_1c_; **(H)** INS. The values are expressed as means ± SD (*n* = 5). Data followed by the different letters are significantly different (*p* < 0.05) by Duncan’s test.

After 4 weeks high fat diet feeding, a significant decline on body weight was found in the RQP group mice, when compared with the HD group (Figure 5A). Additionally, had decreased of epididymal fat and mesenteric fat, and increased brown fat were found in the RQP group mice, when compared with the HD group (Figures 5B,C).

The diabetic mice in the HD group showed a significant increase in blood glucose and HbA_1c_ levels (Figures 5D,G,H). However, this elevation was inhibited by treatment with RQP. In Figure 5H, RQP reduced insulin level compared with the HD group, which indicated that RQP may be able to minimize the stress caused by hyperglycemia. As shown in Figure 5E, The blood glucose levels in the HD and RQP groups peaked 30 min after loading glucose. Additionally, at each time point, the HD group’s blood glucose level was higher than that of the other groups. The AUC’s fluctuations throughout the OGTT are depicted in Figure 5F. The model group responded to oral glucose delivery with a considerable hyperglycemic response. When compared to the HD group, the AUC of the blood glucose response was considerably lower in the RQP group.

#### 3.3.2 Effects of RQP on lipid levels

**Figure 6:**
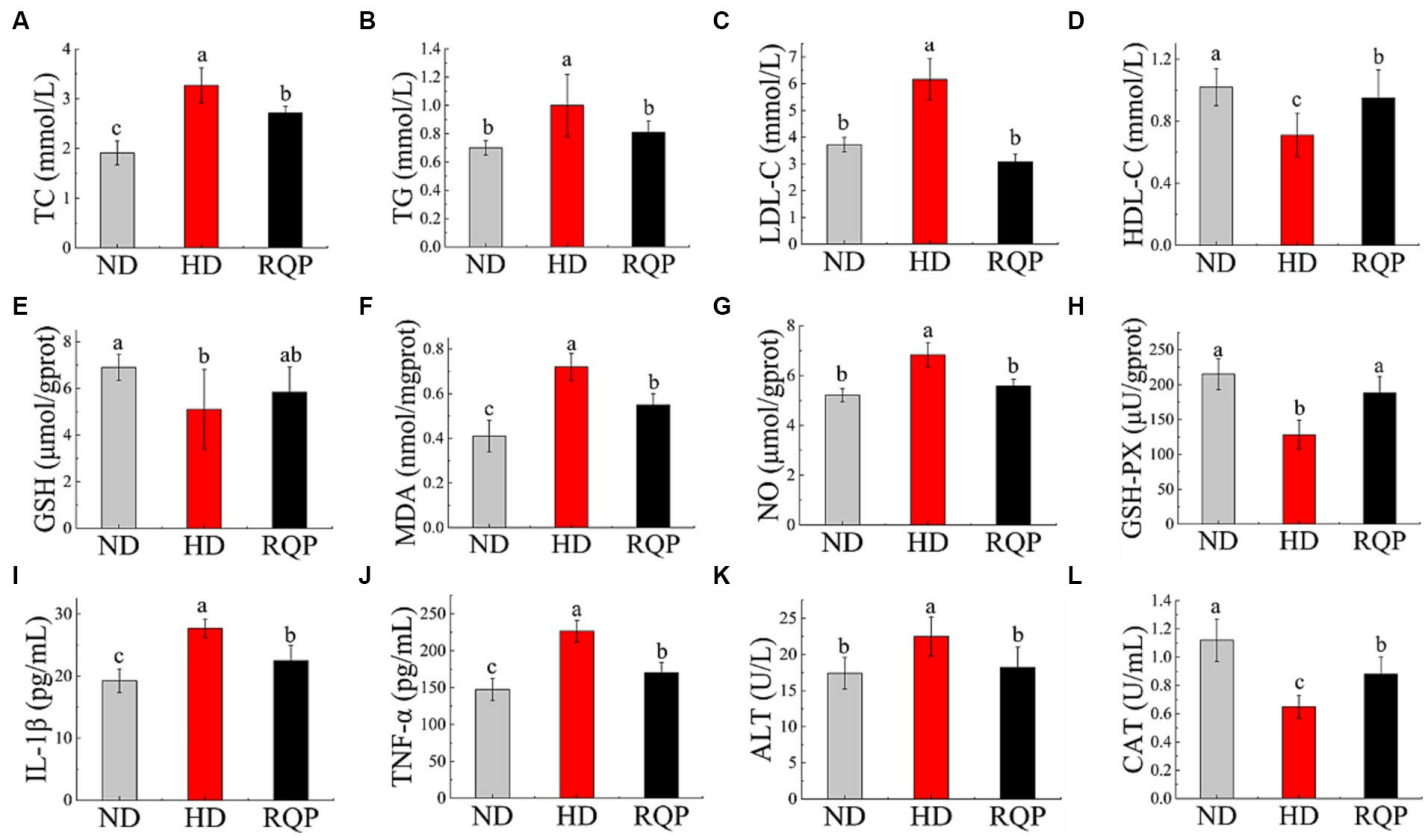
Effects of RQP on lipid level, antioxidant levels and inflammatory factors. **(A)** TC; **(B)** TG; **(C)** LDL-C; **(D)** HDL-C; **(E)** GSH; **(F)** MDA; **(G)** NO; **(H)** GSH-PX; **(I)** IL-1β; **(J)** TNF-α; **(K)** ALT; **(L)** CAT. The values are expressed as means ± SD (*n* = 5). Data followed by the different letters are significantly different (*p* < 0.05) by Duncan’s test.

TC, TG, and LDL-C levels were significantly lower in the RQP group compared with the HD group (Figures 6A–C). The TC content in the RQP group decreased by 16.82%, the TG content decreased by 19.0%, and the LDL-C content decreased by 50.0%. And as shown in Figure 6D, the HDL-C content in the RQP group was significantly increased, which was comparable to that of healthy mice.

#### 3.3.3 Effects of RQP on antioxidant levels in liver

Figures 6E,H show that the GSH-PX content was significantly reduced, but there was no significant difference in GSH content in the RQP group when compared with the HD group. Figure 6G shows that the NO content of RQP was significantly lower than that of HD group. Lipid peroxidation produces MDA, which elevated levels can cause inflammation, necrosis, and damage to cell membranes (Yuan et al., 2018). Figure 6F demonstrates that the MDA level in the HD group was substantially higher than that in the ND group, while it decreased significantly in the RQP group. These findings showed that RQP had a significant impact on the antioxidant levels in diabetic mice.

#### 3.3.4 Effects of RQP on inflammatory factors

TNF-α and IL-1β levels (Figures 6I,J) in the RQP group were significantly decreased compared with the HD group. In addition, the ALT content significantly decreased by while CAT significantly increased in the RQP group (Figures 6K,L), respectively. The results showed that RQP has the potential to improve liver cell injury.

#### 3.3.5 Effects of RQP on SCFAs

**Figure 7:**
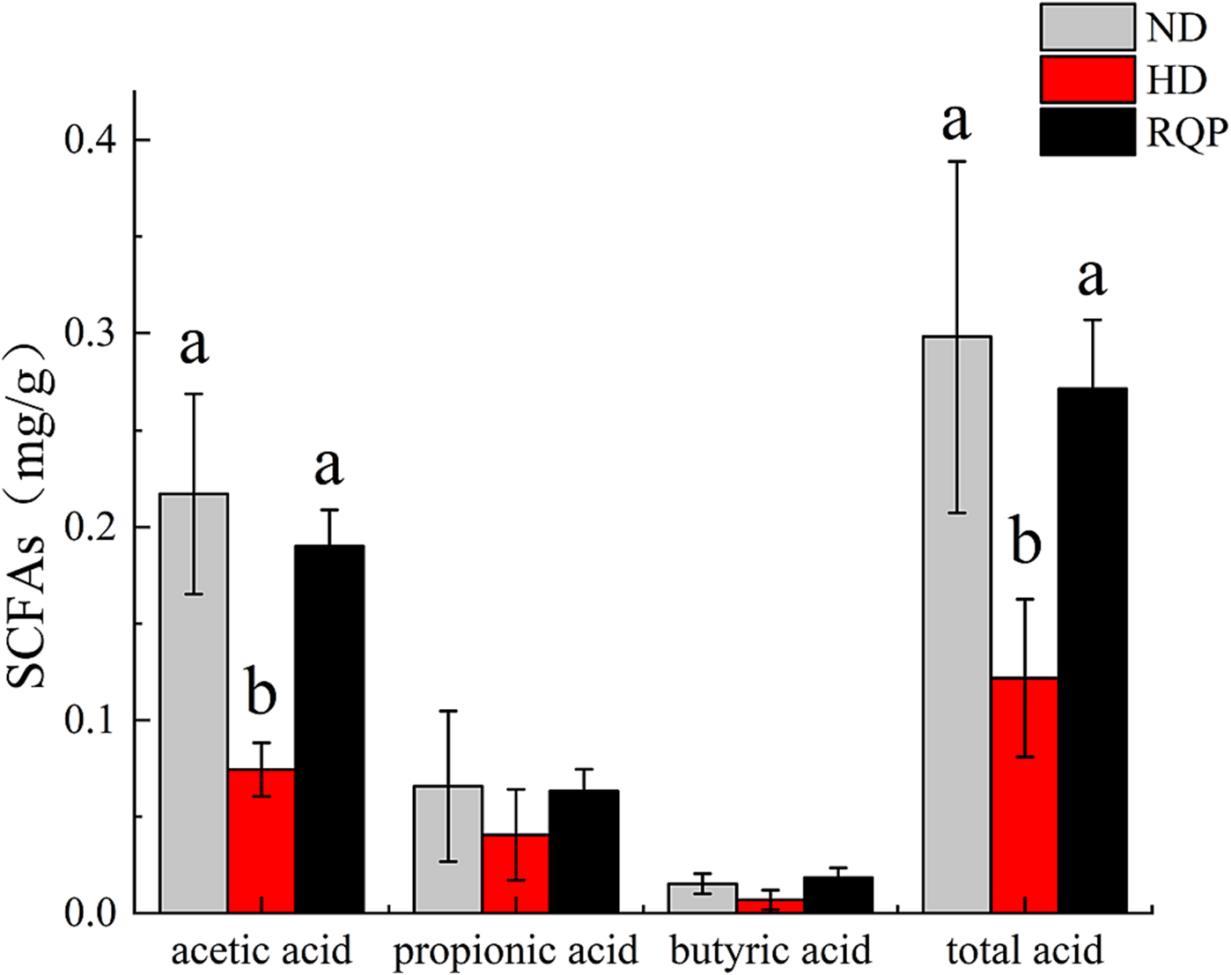
Effects of RQP on SCFAs of C57BL/6 J mice. The values are expressed as means ± SD (*n* = 5). Data followed by the different letters are significantly different (*p* < 0.05) by Duncan’s test.

When compared to the HD group, the concentrations of acetic acid and total acid in the cecal contents of RQP increased significantly, while propionic acid and butyric acid levels increased slightly (Figure 7). The results showed that feeding RQP increased SCFAs levels, which had a potential effect on improving intestinal function of diabetic mice.

#### 3.3.6 Effects of RQP on diversities of gut microbiota

**Figure 8:**
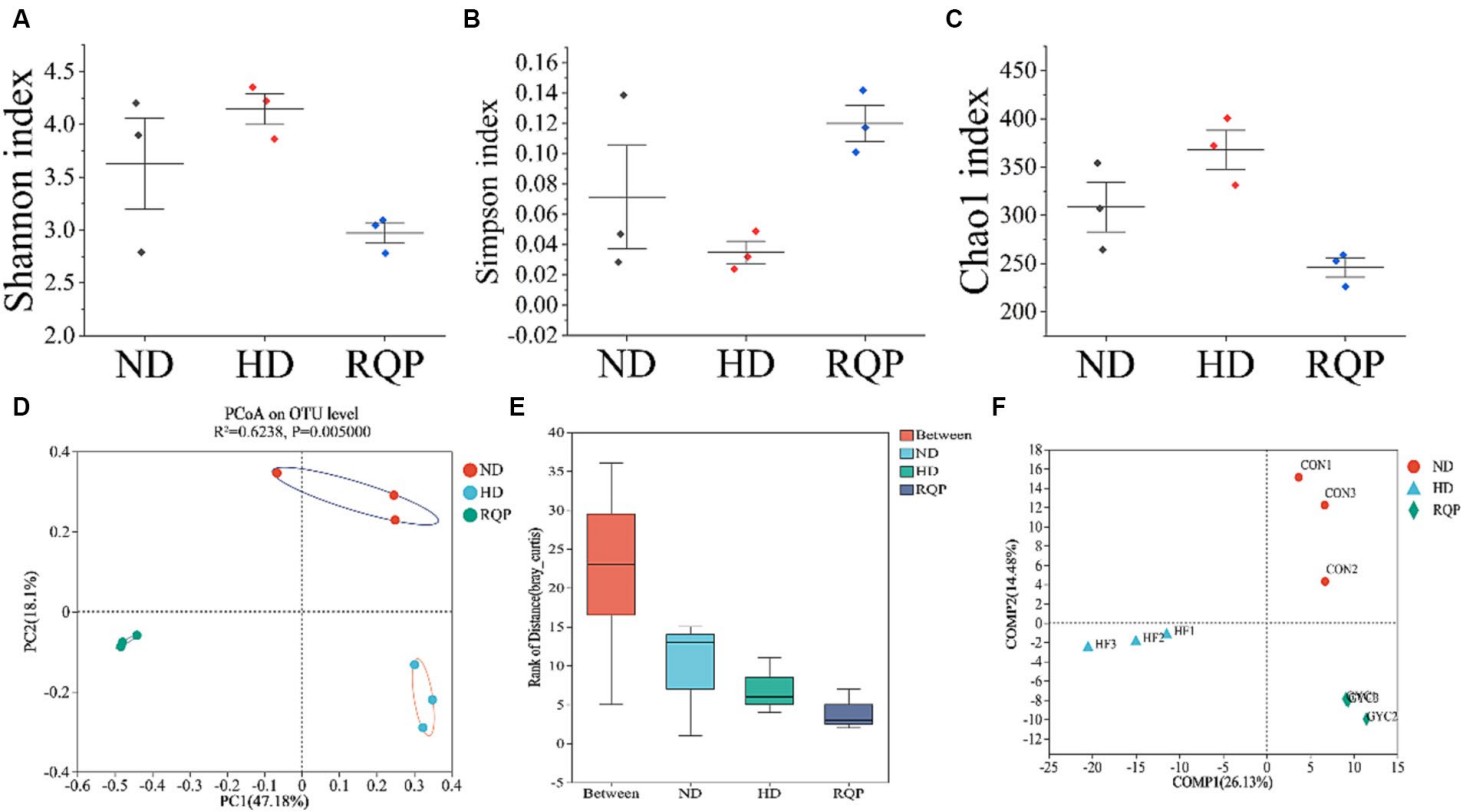
Effects of RQP on the diversity of gut microbiota in diabetic mice. **(A)** Shannon index; **(B)** Simpson index; **(C)** Chao1 index; **(D)** Principal co-ordinates analysis (PCoA); **(E)** Analysis of similarities (ANOSIM); **(F)** Venn analysis. Data are presented as the mean ± SD (*n* = 3).

According to the findings, fecal samples from the ND, HD, and RQP groups were chosen for 16S rRNA sequencing to examine the variations in gut microbiota. Figures 8A–C demonstrate that the RQP group’s microbial community’s species diversity and abundance were lower than those of the HD group. For β-diversity, PCoA analysis, analysis of similarities (ANOSIM) and PLS-DA analysis were performed (Figures 8D–F). The results demonstrated the existence of three distinct clusters within the microbial community, each of which contained a concentrated distribution of samples. Both high-fat diet and RQP had a significant effect on gut microbiological composition, which was demonstrated by more significant between-group changes than within-group differences (*p* < 0.05).

**Figure 9:**
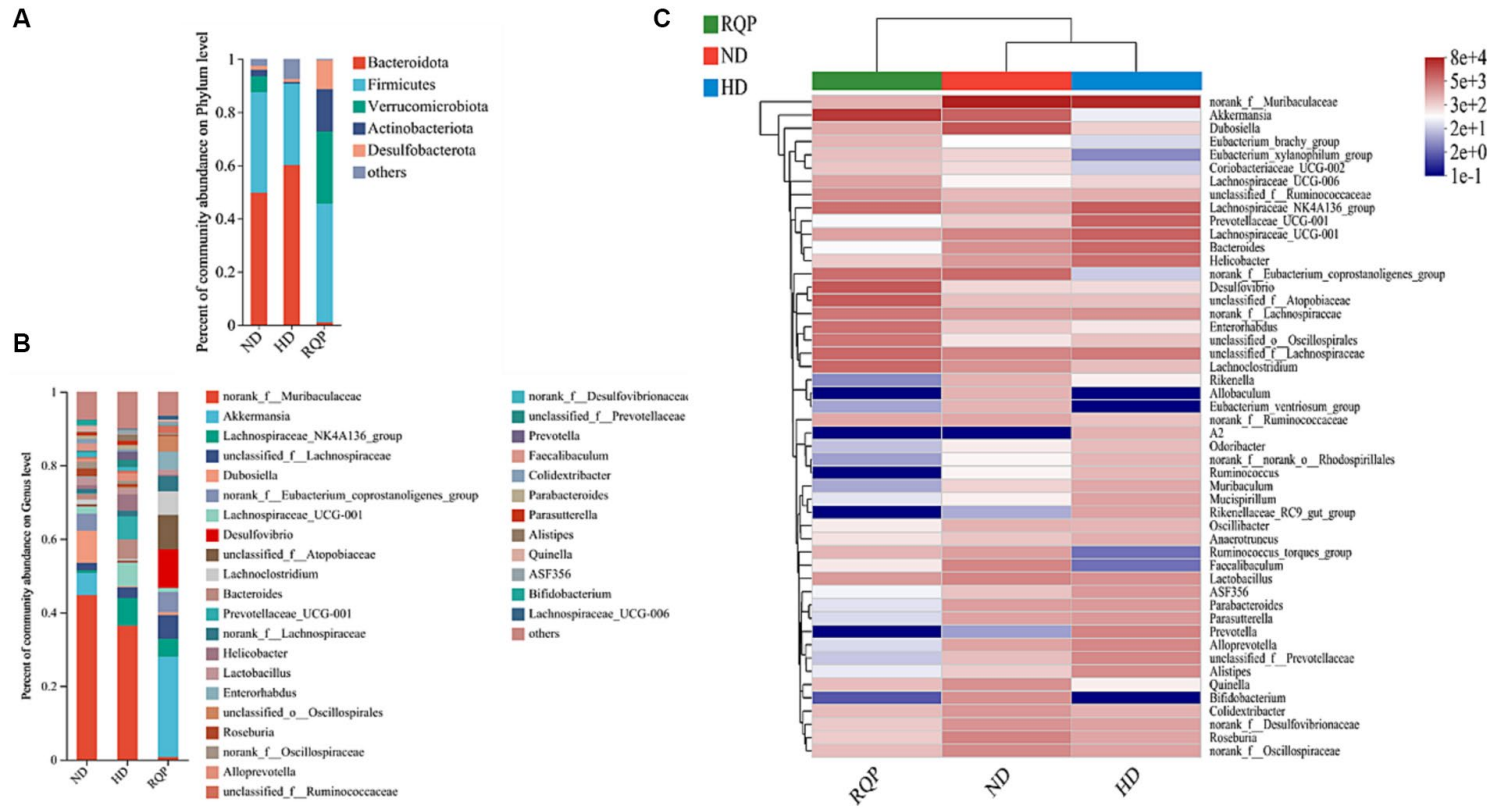
Effects of RQP on the gut microbiota composition in diabetic mice. **(A)** The relative abundance of gut microbiota at the phylum level; **(B)** genus level; **(C)** Community heatmap analysis on genus level. Data are presented as the mean ± SD (*n* = 3).

The relative abundances of several bacterial taxa in each sample were counted at the phylum and genus levels to determine the impact of RQP on the make-up of the bacterial community. In the RQP group, the composition of the gut microbiota considerably changed at the phylum and genus levels (Figure 9A). Compared with the ND group, the *Bacteroidota* level increased and the *Firmicutes*, *Verrucomicrobiota*, *Actinobacteriota* levels were decreased in the HD group. However, compared to the HD group, the RQP group had significantly higher abundances of *Firmicutes*, *Verrucomicrobiota*, *Actinobacteriota*, and *Desulfobacterota*, and significantly decreased abundances of *Bacteroidota*. As shown in Figure 9B at genus level. After RQP treatment, the abundance of *norank_f_Muribaculaceae* decreased from 36.38% to 0.71% as well as the prevalence of the *Lachnospiraceae_NK4A136_group* from 7.49% to 4.93%. On the other hand, the abundances of *unclassified_f_Lachnospiraceae* increased from 2.95% to 6.38%, while the abundances of *Akkermansia* increased to 27.23%. Additionally, *norank_f_Lachnospiraceae*, *unclassified_f_Atopobiaceae*, and *norank_f_Eubacterium_coprostanoligenes_group* in the RQP group were significantly higher than those in the HD group. The results showed that RQP directly impacted the intestinal microbiota of diabetic mice. For further understanding the differences in intestinal flora at the genus level, heat map analysis was performed on the ND, HD, and RQP groups (Figure 9C). *Verrucomicrobia* decreased, because of RQP’s enhanced *Akkermansia* abundance in comparison to the HD group. RQP inhibited the increase of harmful intestinal flora in diabetes mice by reducing the abundance of *A2*, *Odoribacter*, *norank_f_norank_o_Rhodospirillales*, *Ruminococcus*, *Muribaculum*, *Mucispirillum*, *Rikenellaceae_RC9_gut_group*, *Parabacteroids*, *Parasutterella*, *Prevotella*, *Alloprevotella*, *unclassified_f_Prevotellaceae* and *Alistipes*. In addition, the abundance of some beneficial bacteria increased after RQP treatment, such as *Eubacterium_brachy_group*, *Eubacterium_xylanophilum_group*, *Coriobacteriaceae UCG-002*, *Ruminococcus_torques_group* and *Faecalibaculum*.

**Figure 10:**
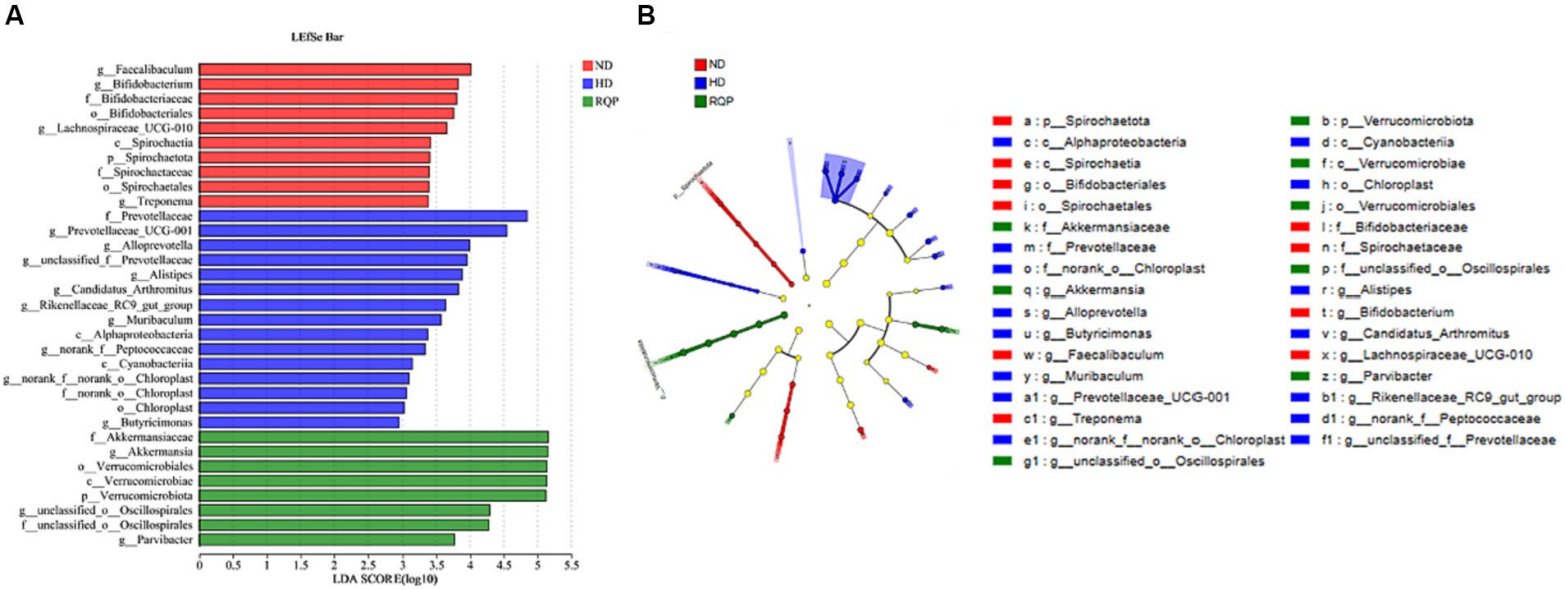
Core bacterial phenotype analysis in in diabetic mice (*n* = 3). **(A)** LDA scores of taxa enriched at different taxonomy levels (LDA significant threshold = 4); **(B)** Taxonomic cladogram generated by LEfSe analysis.

In addition, LEfSe analysis highlights the core bacterial phenotype from phylum to genus, to understand the changes in microbial composition. As shown in Figures 10A,B, there was no significant difference among the three groups in *Proteobacteria*, *Bacteroidota*, *Firmicutes*, *Actinobacteriota*, *Cyanobacteria*. However, the ND group were enriched with the phylum *Spirochaetota*, the class *Spirochaetia*, the order *Spirochaetales* and *Bifidobacteriales*, the family *Spirochaetacea* and *Bifidobacteriaceae*, the genus *Treponema*, *Spirochaetia*, *Spirochaetales*, *Spirochaetaceae*, *Bifidobacteriales*, *Bifidobacteriaceae*, *Bifidobacterium*, *Faecalibaculum* and *Lachnospiraceae_UCG-010*. The HD group were enriched with the class *Cyanobacteriia* and *Alphaproteobacteria*, the order *Chloroplast*, the family *Chloroplast* and *Prevotellaceae*, the genus *Cyanobacteriia*, *Chloroplast*, *f_norank_o_Chloroplast*, *g_Prevotellaceae_UCG-001*, *Alphaproteobacteria*, *g_Prevotellaceae_UCG-001*, *f_Prevotellaceae*, *g_Alloprevotella*, *g_unclassified_f_Prevotellaceae*, *g_Butyricimonas*, *g-Muribaculum*, *g_Alistipes*, *g_Rikenellaceae_RC9_gut_group*, *g_Candidatus_Arthromitus*, *g_norank_f_Prevotellaceae*. The above results showed that the intestinal flora of mice fed with high-fat diet changed significantly. The gut microbiota enriched in the RQP group were the phylum *Verrucomicrobiota*, the class *Verrucomicrobiae*, the order *Verrucomicrobiales*, the family *Akkermansiaceae* and *f_unclassified_o_Oscillospirales*, the genus *f_Akkermansiaceae*, *p_Verrucomicrobiota*, *c_Verrucomicrobiae*, *o_Verrucomicrobiales*, *f_unclassified_o_Oscillospirales*, *g_Akkermansia*, *g_Parvibacter*, *g_unclassified_o_Oscillospirales*.

**Figure 11:**
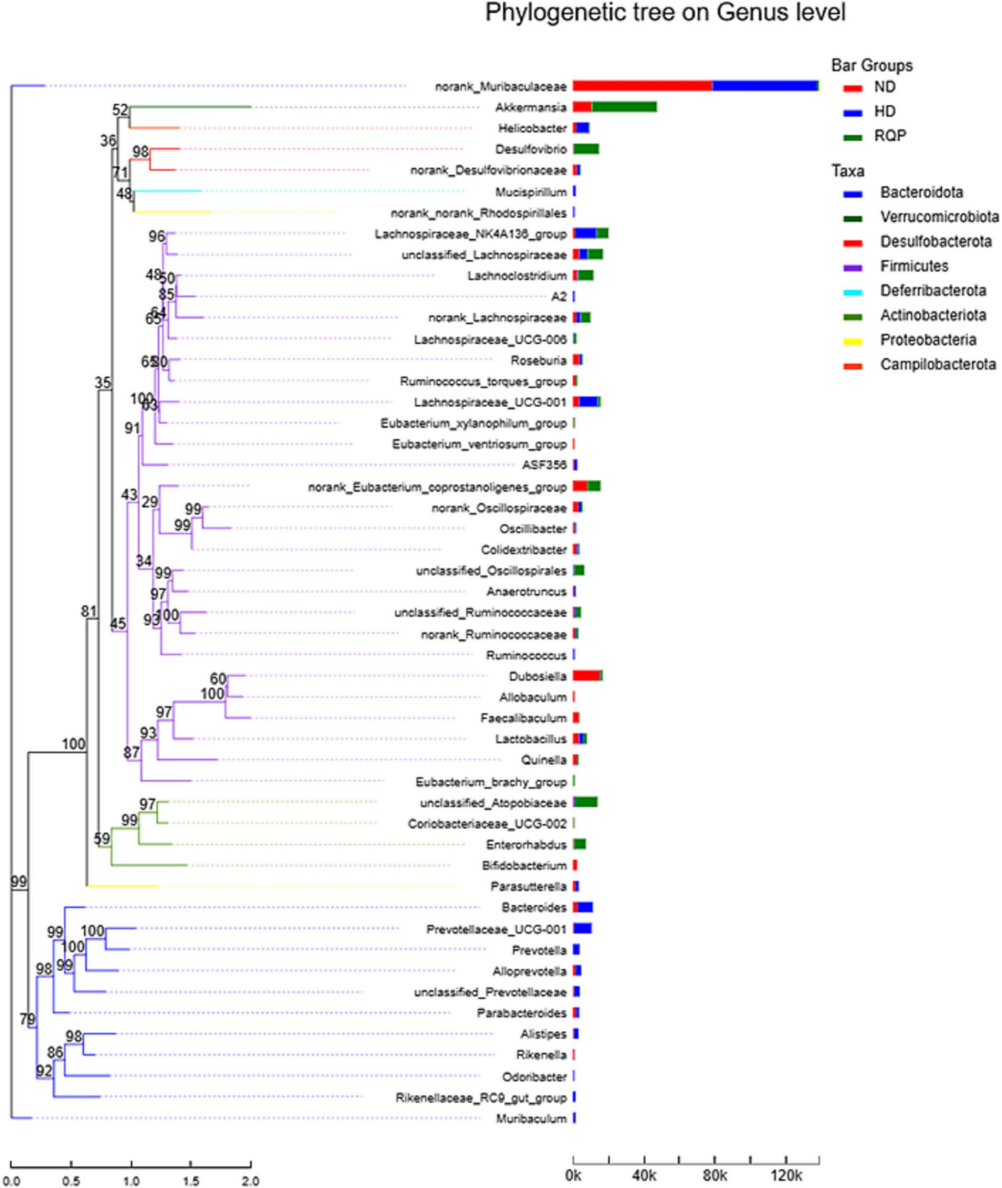
Phylogenetic tree on Genus level.

According to the evolutionary relationship among the species in the sample, the phylogenetic tree was constructed to reveal the genetic relationship of the species in the sample during the evolution process from the perspective of molecular evolution. As shown in Figure 11, there are significant differences in the evolutionary distance between the bacteria, especially after RQP treatment. The results showed that RQP could change the disorders of intestinal microbiota in diabetic mice induced by high-fat diet.

**Figure 12:**
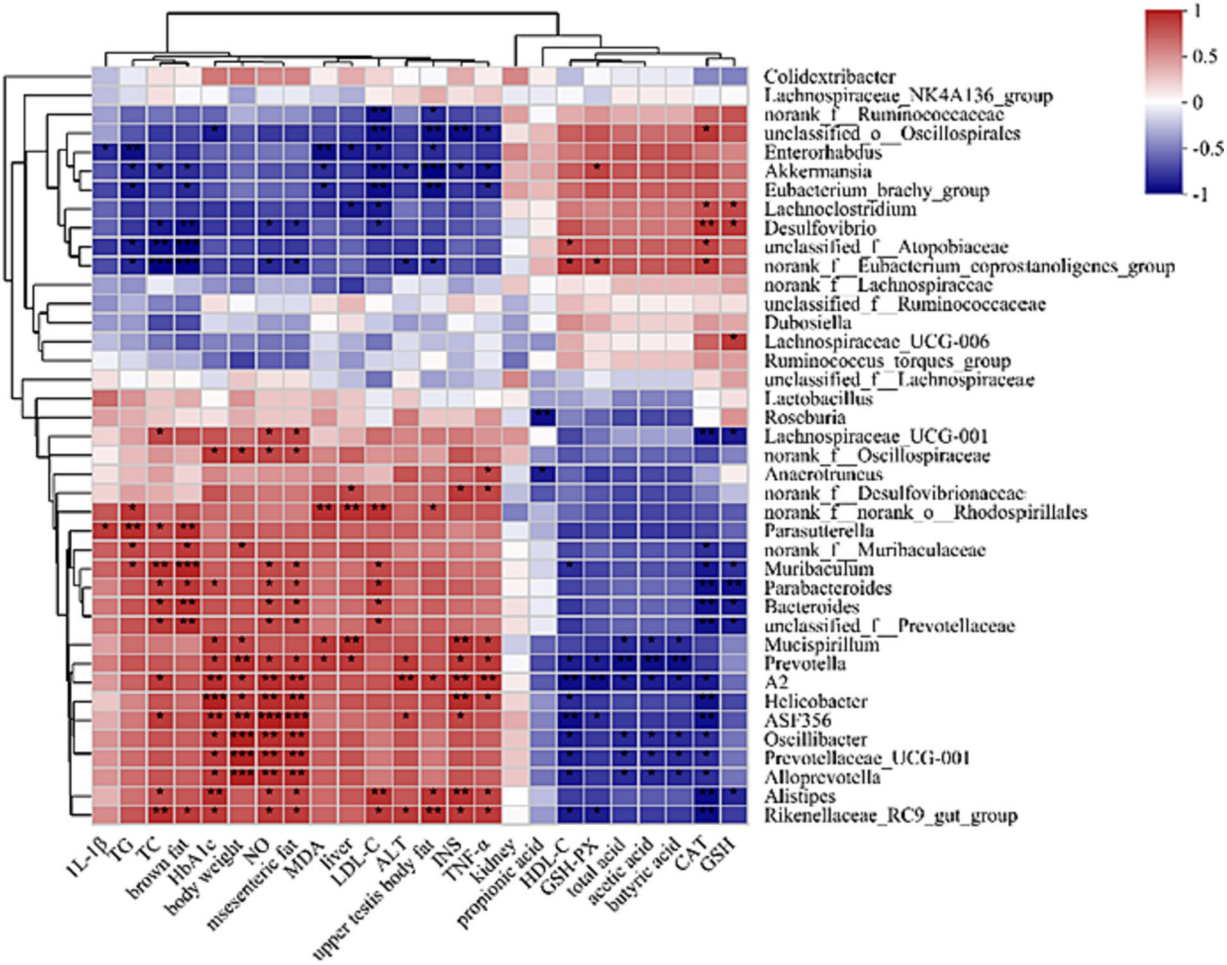
Spearman correlation heatmap between gut microbes and physiological indicators in mice at the genus level.

To determine if RQP-induced alterations in the composition of the gut microbiota and the amounts of biochemical indicator molecules might be related, we used Spearman’s correlation analysis (Figure 12). Specifically, *Akkermansia* are significantly positively correlated with GSH-PX, negatively correlated with TNF-α, INS, ALT, LDL-C, ALT, MDA, TC, TG, brown fat and upper testis body fat. *Mucispirillum*, *Prevotella*, *A2*, *Helicobacter*, *ASF356*, *Oscillibacter*, *Prevotellaceae_UCG-001*, *Alloprevotella* are significantly positively correlated with HbA_1c_, body weight, NO and mesenteric fat, negatively correlated with SCFAs.

#### 3.3.7 BugBase phenotype prediction

**Figure 13:**
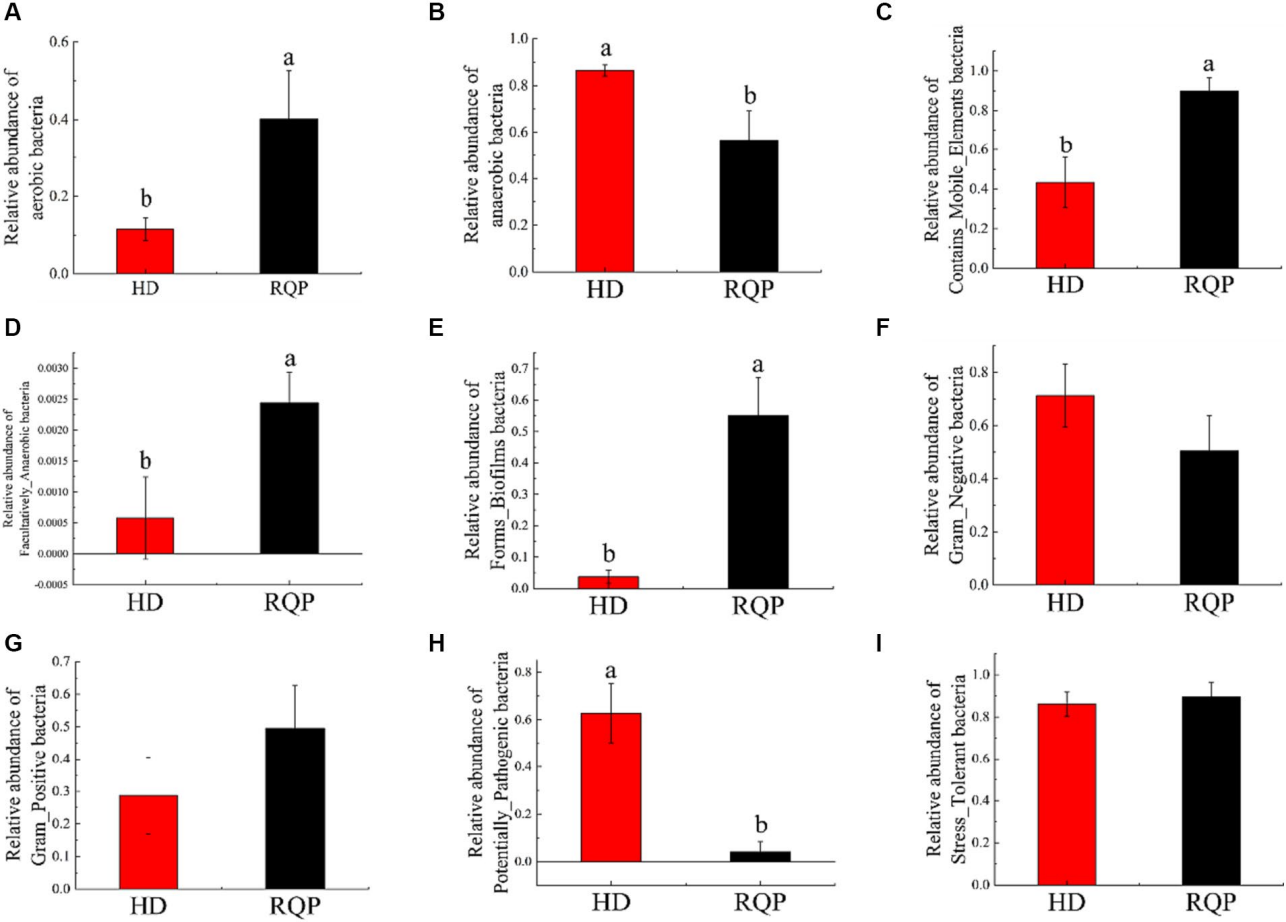
Effects of RQP on microbiome phenotypes. **(A)** aerobic bacteria; **(B)** anaerobic bacteria; **(C)** Contains_Mobile_Elements bacteria; **(D)** Facultatively_Anaerobic bacteria; **(E)** Forms_Biofilms bacteria; **(F)** Gram_Negative; **(G)** Gram_Positive; **(H)** Potentially_Pathogenic; **(I)** Stress_Tolerant. Data are presented as the mean ± SD (*n* = 3). Data followed by the different letters are significantly different (*p* < 0.05) by Duncan’s test.

The phenotype of the microbiome was studied. The relative abundance of aerobic bacteria and mobile elements bacteria is depicted in Figure 13, Facultatively_Anaerobic bacteria and Forms_Biofilms were lower. In contrast, Anaerobic bacteria, and microorganisms with potential for disease were more prevalent overall. However, the situation was the opposite in the RQP group. The relative abundance of Gram_Negative, Gram_Positive and Stress_Tolerant were not significantly difference between HD and RQP group. Therefore, RQP can also affect the composition of intestinal microorganisms by changing bacterial phenotypes.

#### 3.3.8 PICRUSt1 and FAPROTAX function prediction

**Figure 14:**
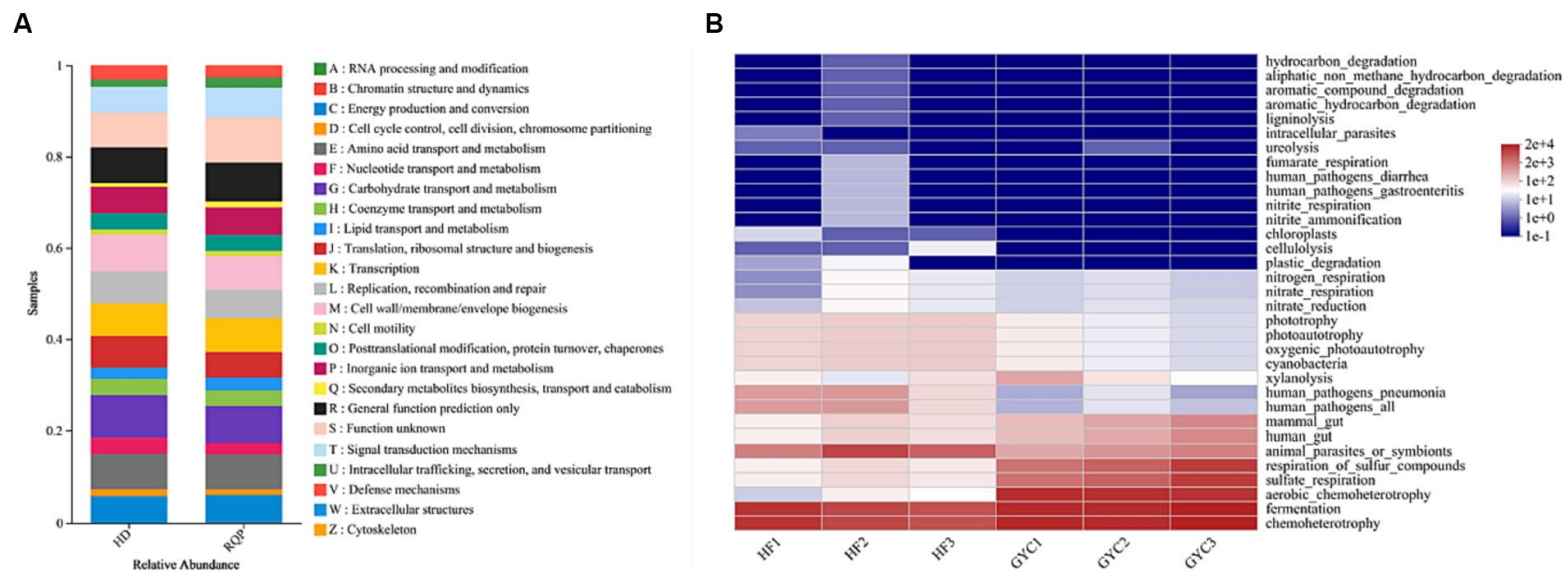
PICRUSt1 and FAPROTAX function prediction. **(A)** PICRUSt1 function prediction results stacked bar chart; **(B)** The prediction of intestinal microbiota function between HD and RQP by heatmap analysis.

PICRUSt1 function prediction can be used for OTU annotation information at the functional level of COG and the abundance information of each function in different samples. FAPROTAX function prediction can be used to predict the metagenomic contribution of intestinal microbiota. As shown in Figure 14A, RQP significantly changed the abundance of OTU in the biosynthetic pathway and metabolic pathway, compared with the HD group. Figure 14B indicated that high-fat diet may lead to the production of potential pathogens, such as human_pathogens_pneumonia and human_pathogens_all. RQP can effectively inhibit the production of these two bacteria. The above experimental results further indicate that RQP can alleviate intestinal flora disorders and related metabolic dysfunction.

## 4 Discussion

In this study, we evaluated the hypoglycemic, hypolipidemic, and gut microbiota effects of RQP in diabetic mice. According to HPLC data, RQP in this study had a yield of 1.57 ± 0.38% and was a complex glycoconjugate. RQP is primarily composed of Man (2.17%), Rib (1.76%), Rha (1.37%), GlcA (0.90%), GalA (1.21%), Glu (64.55%), Gal (13.22%), Xyl (0.54%), Ara (13.15%), Fuc (1.04%). These findings are in line with the previous studies on RQP, and Glu, Gal, and Ara are prevalent in it (Tan et al., 2021). The results of monosaccharide composition, FITR and HPLC preliminarily revealed the composition and basic structure of RQP, which provided a framework for future research on the functional activity of RQP.

RQP was proved good antioxidant activities *in vitro*. Compared with O_2_^−^ and ·OH, RQP showed higher antioxidant effects on DPPH and ABTS radicals, which may be due to the different antioxidant systems and different antioxidant mechanisms (Huang et al., 2017; Mu et al., 2021). The good antioxidant activities may be connected with low molecular weight of polysaccharides, which contributed by ultrasound-assisted extraction, and reported to be easier to transfer electrons from uronic acid groups (Huang and Huang, 2020) Another explanation is RQP contains high proportion of glucose, acarbose, and galactose, which was proved to contribute to higher effects on DPPH and ABTS scavenging (Huang and Huang, 2020). Inhibitors of α-amylase and α-amylase were essential for delaying human carbohydrate absorption and reducing the release of glucose from carbohydrates (Lv et al., 2021). A previous study has shown that the arabinose and xylose content of polysaccharides determines the inhibitory effect of polysaccharides on α-glucosidase and α-amylase (Lv et al., 2021). In the present study, the arabinose and xylose contents of RQP were 13.15% and 0.54%, respectively. This could be the reason for the lower inhibition of α-amylase and α-glucosidase by RQP. In this study, the inhibitory effects on both α-glucosidase and α-amylase may partly contribute to the improvement of hyperglycemia and hyperlipidemia in mice (Zhang et al., 2019). To function as prebiotics, polysaccharides must pass through the digestive tract and saliva to reach the colon. Previous studies have shown that polysaccharides are not hydrolyzed by digestive enzymes in saliva, gastric juice, and small intestinal fluid, which are consistent with our findings (Ding et al., 2019; Ma G. et al., 2022).

T2D is a persistent medical condition distinguished by elevated blood glucose levels, often accompanied by cardiovascular disease, renal disease, eye disease, and other consequences. These complications arise from the impaired functionality of islet β cells within the body, leading to insulin resistance (Chatterjee et al., 2017). Long-term intake of high-fat diet can lead to weight gain, fat accumulation and elevated blood glucose in diabetic mice, resulting in insulin resistance, which makes the mice endocrine more insulin to maintain normal glucose and lipid metabolism in the body (Luo et al., 2021). We found that body weight, organ index, and FBG were significantly decreased in diabetic mice after 4 weeks RQP treatment. These results indicated that RQP effectively regulates glycolipid metabolism in an *in vivo* setting by enhancing glucose absorption and utilization in mice with T2D. The average blood glucose levels of the three groups of mice during the experiment were indicated by the OGTT and HbA_1c_ readings (Liu et al., 2018). Compared with the HD group, RQP improved OGTT and significantly reduced HbA_1c_ level in diabetes mice, indicated that the intake of RQP improved insulin resistance, helps maintain blood sugar homeostasis in the body (Yang et al., 2021). Disorders of lipid metabolism are another prominent feature of diabetes (Kane et al., 2021). Following consumption of a high-fat diet, the mice mostly displayed an increase in serum levels of TC, TG, LDL-C, while exhibiting a decrease in HDL-C (Athyros et al., 2018). This study found that RQP significantly improved the dyslipidemia caused by high fat-diet, decreased the contents of serum TC, TG, LDL-C, and increased HDL-C of diabetic mice. The findings suggest that RQP could modulate lipid metabolism and concurrently preserve blood glucose stability, hence exerting a partial inhibitory effect on diabetic outcomes.

Previous studies have indicated a strong association between oxidation and diabetes, whereby oxidative stress is heightened, hence exacerbating the advancement of T2D and its associated consequences (Rehman and Akash, 2017; Yaribeygi et al., 2020). Prolonged consumption of a high-fat diet can lead to an alteration in the equilibrium of free radicals inside the human body, leading to an excessive generation of free radicals and subsequently triggering a reaction of oxidative stress (Pasaoglu et al., 2004). In the context of T2D, it was observed that the high-fat diet administered to mice resulted in an elevation of lipid oxidation, hence contributing to oxidative damage (Yaribeygi et al., 2020). The antioxidative properties of RQP were demonstrated by its capacity to reduce the levels of MDA, NO, and GSH, while simultaneously increasing the levels of GSH-PX and CAT in the bloodstream. Further, significant decreases in IL-1β, TNF-α and ALT activities were also found in mice of the RQP group, which were inflammatory factors and play vital role in the development of insulin resistance in T2D cases (Liu et al., 2016). The findings of this study indicated that the treatment of the RQP has the potential to mitigate oxidative damage and inflammatory levels resulting from hyperglycemia and hyperlipidemia.

Based on the above findings, this experiment further found that RQP intake significantly increased the content of SCFAs. SCFAs are important metabolites that regulate intestinal inflammation and metabolism, which were produced by microorganisms and difficult-to-digest polysaccharides (Sivaprakasam et al., 2016). SCFAs have the capacity to influence glucose homeostasis and modulate blood glucose levels through their regulatory effects on glucose absorption and utilization inside the human body (Rauf et al., 2022). Polysaccharides are fermented by the intestinal flora in the large intestine, where they are converted into different short-chain fatty acids due to their different structures and monosaccharide composition (Zhang et al., 2020). Acetic acid is produced mainly by the fermentation of glucuronic acid, xylose, galactose, and galacturonic acid, propionic acid is produced by the fermentation of glucose, xylose, and arabinose, and butyric acid is produced from xylose, glucuronic acid, galactose, and galacturonic acid. Acetate, propionate, and butyrate serve as metabolic substrates in the process of cholesterol production (Wang et al., 2019). In addition, propionate can also inhibit the synthesis of cholesterol and fatty acids in the liver (Portincasa et al., 2022). In this study, RQP increased the content of acetic acid and total acid compared with the HD group, indicated that RQP could improve the intestinal function of diabetic mice by increasing the contents of SCFAs, thereby regulating lipid metabolism, reducing blood glucose and lipid accumulation (Portincasa et al., 2022).

There is a growing body of evidence supporting the significant involvement of gut bacteria in the development and progression of lipid metabolic diseases (Ma et al., 2019). The composition of gut microbiota plays a crucial role in the manifestation and progression of metabolic syndrome through its impact on host energy metabolism, immune system function and inflammatory response (Wu et al., 2021). We investigated the regulatory effect of RQP on intestinal microflora by comparing special bacteria. The results showed that RQP intervention reversed the abundance of Bacteroidetes and Firmicutes, indicated that it could improve the proportion of bacteria in the body, inhibit the growth of some pathogenic bacteria and reduce inflammation (Ma Q. et al., 2022). Our study also found that *Akkermansia* was the dominant bacteria after RQP intervention, which was reported to produce SCFAs in the metabolic process, and expected to be a potential target for improving metabolic diseases such as obesity, diabetes, liver disease and cardiac metabolic disorders (Rodrigues et al., 2022). Another study showed that *Akkermansia* can also produce *Amuc _ 1100*, *P9* and other proteins which directly involved in the regulation of glucose and lipid metabolism and immune response, while as stimulates glucagon-like peptide 1 (GLP-1) secretion by producing short-chain fatty acids such as acetic acid and propionic acid to regulate host energy balance and improve glucose homeostasis in mice (Yoon et al., 2021). In this study, increased *Akkermansia* may be one of the reasons that RQP intervention, could regulate glycolipid metabolism and inhibit the development of diabetes.

This study employed the PICRUSt1 approach to assess the abundance of OTUs at the functional level. Additionally, the BugBase analytic method was utilized to investigate the impact RQP on microbial phenotype. After RQP treatment, the abundance of biosynthetic and metabolic pathways was change, such as carbohydrate transport and metabolism and translation, ribosomal structure, and biogenesis. The observed decline in the abundance of anaerobic bacteria and potentially pathogenic bacteria inside the intestinal tract of the RQP group suggests that RQP has the capacity to mitigate the quantity of pathogenic bacteria and promote the restoration of intestinal health. The above results indicate that RQP can improve the biosynthetic pathway in diabetic mice, reduce intestinal metabolic disorders in mice, and reverse the potential risks of related pathogens.

## 5 Conclusion

In this study, RQP is a complex polysaccharide composed of a variety of monosaccharides, which has good antioxidant activity and α-amylase and α-glucosidase inhibitory activity *in vitro*. At the same time, this study indicated that RQP showed effective antioxidant and hypoglycemic activity in obese diabetic mice and regulated the structure of intestinal flora. Specifically, the supplementation of RQP reduced the weight gain of obese mice feed with a high-fat diet, which has regulated lipid metabolism and inhibited insulin resistance to reduce blood glucose. RQP also improved the antioxidant capacity of the body reduced inflammation and promoted the production of SCFAs in diabetic mice. Furthermore, structural alterations of the gut microbiota induced by high-fat diet were modulated by RQP, which would be one of the important hypoglycemic lipid-lowering mechanism. Our findings indicate that polysaccharides from red quinoa may possess potential health properties in alleviating the symptoms associated with T2D.

## Data availability statement

The original contributions presented in the study are included in the article/Supplementary material, further inquiries can be directed to the corresponding authors.

## Ethics statement

The animal study was approved by Experimental Animals Ethics Committee of Heilongjiang Bayi Agricultural University. The study was conducted in accordance with the local legislation and institutional requirements.

## Author contributions

YZ: Conceptualization, Funding acquisition, Project administration, Resources, Writing – review & editing. YG: Formal analysis, Investigation, Software, Validation, Visualization, Writing – original draft. YC: Project administration, Supervision, Writing – review & editing. HT: Project administration, Supervision, Writing – review & editing.
