# Supplementary material for: Anti-diabetic effect of red quinoa polysaccharide on type 2 diabetic mellitus mice induced by streptozotocin and high-fat diet
Source: Front Microbiol. 2024 Feb 27;15:1308866. doi: 10.3389/fmicb.2024.1308866 (PMC10929017; doi:10.3389/fmicb.2024.1308866)
Supplement: Supplementary file 1 [file Table_1.DOCX]

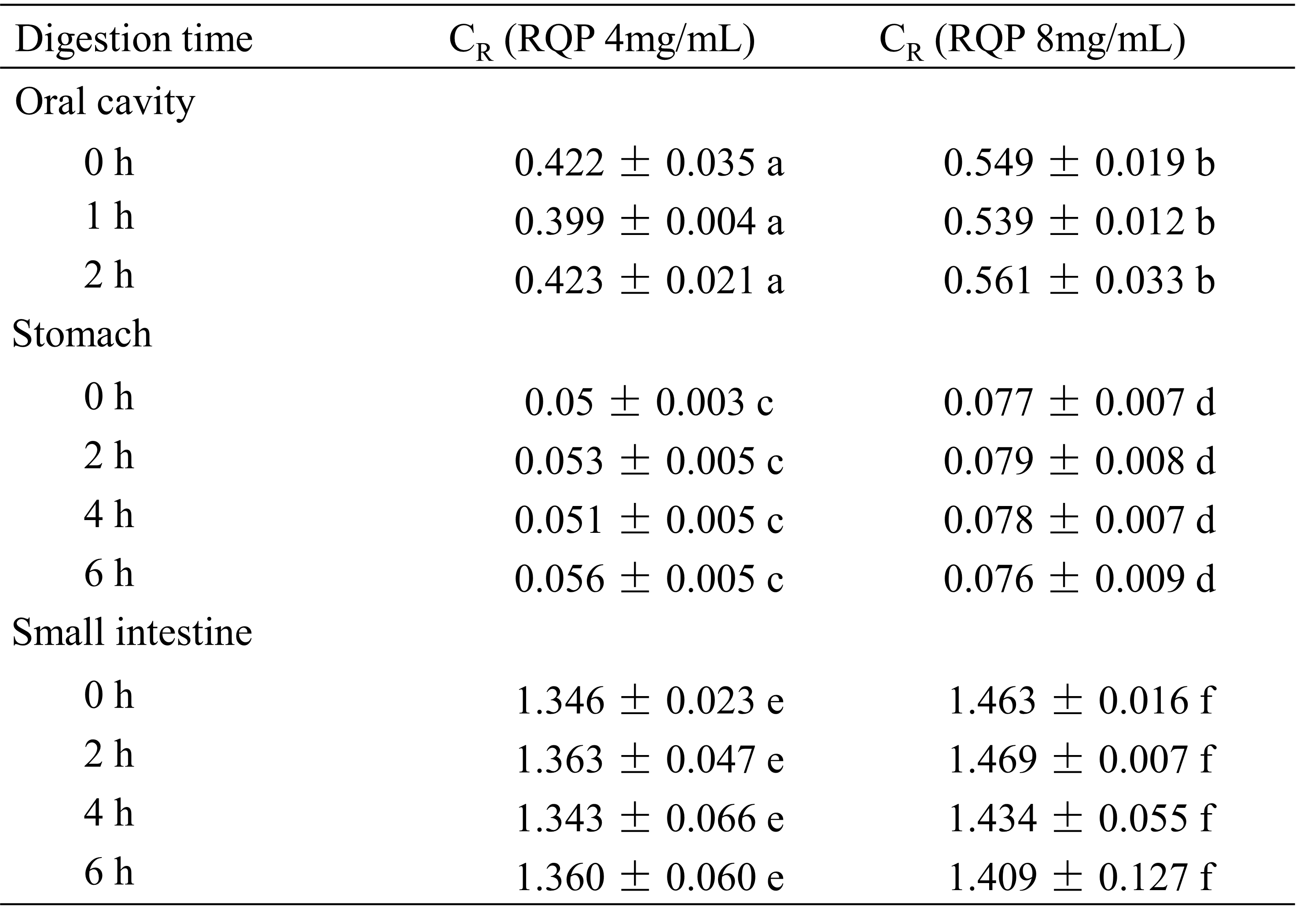
**Suppl. Table. S1** In vitro digestion

The values are expressed as means ± SD (n=3). Data followed by the different letter are significantly different (*P* < 0.05).
